# Doxorubicin-Induced Autophagolysosome Formation Is Partly Prevented by Mitochondrial ROS Elimination in DOX-Resistant Breast Cancer Cells

**DOI:** 10.3390/ijms22179283

**Published:** 2021-08-27

**Authors:** Seyedeh Tayebeh Ahmadpour, Valérie Desquiret-Dumas, Ulku Yikilmaz, Julie Dartier, Isabelle Domingo, Celine Wetterwald, Charlotte Orre, Naïg Gueguen, Lucie Brisson, Karine Mahéo, Jean-François Dumas

**Affiliations:** 1 Inserm UMR1069 Nutrition, Croissance et Cancer, Université de Tours, 37032 Tours, France; ulku.yikilmaz@etudiant.univ-rennes1.fr (U.Y.); julie.dartier@gmail.com (J.D.); isabelle.domingo@univ-tours.fr (I.D.); lucie.brisson@univ-tours.fr (L.B.); karine.maheo@univ-tours.fr (K.M.); 2 MitoLab Team, Institut MitoVasc, CNRS UMR6015, INSERM U1083, Angers University, 49933 Angers, France; VaDesquiret@chu-angers.fr (V.D.-D.); charlotte.orre@etud.univ-angers.fr (C.O.); nagueguen@chu-angers.fr (N.G.); 3 Department of Biochemistry and Molecular Biology, University Hospital Angers, 49933 Angers, France; sunja@hotmail.fr

**Keywords:** mitochondria, mitophagy, breast cancer, reactive oxygen species, doxorubicin

## Abstract

Since its discovery, mitophagy has been viewed as a protective mechanism used by cancer cells to prevent the induction of mitochondrial apoptosis. Most cancer treatments directly or indirectly cause mitochondrial dysfunction in order to trigger signals for cell death. Elimination of these dysfunctional mitochondria by mitophagy could thus prevent the initiation of the apoptotic cascade. In breast cancer patients, resistance to doxorubicin (DOX), one of the most widely used cancer drugs, is an important cause of poor clinical outcomes. However, the role played by mitophagy in the context of DOX resistance in breast cancer cells is not well understood. We therefore tried to determine whether an increase in mitophagic flux was associated with the resistance of breast cancer cells to DOX. Our first objective was to explore whether DOX-resistant breast cancer cells were characterized by conditions that favor mitophagy induction. We next tried to determine whether mitophagic flux was increased in DOX-resistant cells in response to DOX treatment. For this purpose, the parental (MCF-7) and DOX-resistant (MCF-7dox) breast cancer cell lines were used. Our results show that mitochondrial reactive oxygen species (ROS) production and hypoxia-inducible factor-1 alpha (HIF-1 alpha) expression are higher in MCF-7dox in a basal condition compared to MCF-7, suggesting DOX-resistant breast cancer cells are prone to stimuli to induce a mitophagy-related event. Our results also showed that, in response to DOX, autophagolysosome formation is induced in DOX-resistant breast cancer cells. This mitophagic step following DOX treatment seems to be partly due to mitochondrial ROS production as autophagolysosome formation is moderately decreased by the mitochondrial antioxidant mitoTEMPO.

## 1. Introduction

Mitophagy is a selective form of autophagy in which damaged or excessive mitochondria are degraded, frequently in response to imposed stresses, such as hypoxia and nutrient deprivation [1]. In cancer, mitophagy appears to be tumor-promoting or tumor-suppressive depending on tumor type or stage of progression. Indeed, preserving a basal level of mitophagy facilitates cell survival, but excessive mitophagy may activate apoptosis pathways [2]. For instance, in breast cancer, the most commonly diagnosed cancer in women worldwide, it has been shown that reduced/defective mitophagy promoted tumor growth and metastasis and predicted poor metastasis-free survival [3,4]. On the contrary, data showed activation of mitophagy reduced drug-induced apoptosis in breast cancer cells, suggesting that once a tumor has progressed to an advanced stage, mitophagy may mitigate the treatment-induced stress and participate in the chemoresistance of cancer cells [5,6].

Doxorubicin (DOX) belongs to the group of anthracyclines that are very effective chemotherapeutic agents used in the treatment of a wide range of malignant tumors. In the treatment of breast carcinoma, DOX is one of the most effective drugs in early and late-stage tumors [7]. Several enzymes, including cytochrome P450 reductase, mitochondrial complex 1, and xanthine oxidoreductase, have been shown to reduce DOX via a one-electron reduction mechanism, giving rise to the semiquinone intermediate, which can rapidly reduce oxygen to superoxide (O_2_^•−^). The redox activation of DOX in O_2_^•−^, hydrogen peroxide (H_2_O_2_), and the formation of iron-catalyzed hydroxyl radicals has been suggested as the mechanism of DOX-induced cytotoxicity [8]. Reactive oxygen species (ROS) can also induce mitochondrial damage and mitophagy [9,10,11]. There is now compelling evidence that ROS play an important role in the regulation of hypoxia-inducible factor-1 alpha (HIF-1 alpha) under normoxia and hypoxia [12]. On the other hand, HIF-1 alpha has been shown to induce mitophagy [13]. Some studies suggest that the activation of mitophagy leads to resistance to DOX treatment. For instance, mitophagy mediated by BCL2/adenovirus E1B 19 kDa protein-interacting protein 3 Like (NIX) results in DOX resistance in HCT8 colorectal cancer cells [14]. Interestingly, a combination of DOX and liensinine increases the anti-cancer effect of DOX. This is based on the observation that DOX-mediated apoptosis may be further promoted by liensinine, which inhibits mitophagy by preventing the fusion of the autophagosome with the lysosome [15]. Other studies suggest that inhibition of mitophagy could play a key role in preventing breast cancer incidence [16,17]. The flavonoid TL-2–8 has been shown to prevent breast cancer by blocking mitophagy. Thus, TL-2–8 by blocking the fusion of the mitophagosome with the lysosome improved the survival of mice carrying breast cancer xenografts [17]. Fructose-1,6-bisphosphatase (FBP1), an enzyme limiting the rate of glyconeogenesis, is generally considered to be a suppressor of breast cancer. FBP1 has been shown to induce apoptosis in human breast cancer cells by inhibiting HIF-1α/BNIP3-mediated mitophagy [16]. However, the crosstalk between mitophagy, redox signaling, and HIF-1 alpha is not well understood in the context of DOX resistance in breast cancer cells.

The objective of this study was therefore to explore whether the resistance of breast cancer cells to DOX was associated with conditions that favor mitophagy induction. We particularly investigated the response of mitophagic flux to DOX treatment and ROS production and HIF-1 alpha expression in basal conditions in a DOX-resistant breast cancer cell line model.

## 2. Results

### 2.1. Increased Glycolysis and Dysfunctional Mitochondria in DOX-Resistant Breast Cancer Cells

**Figure 1:**
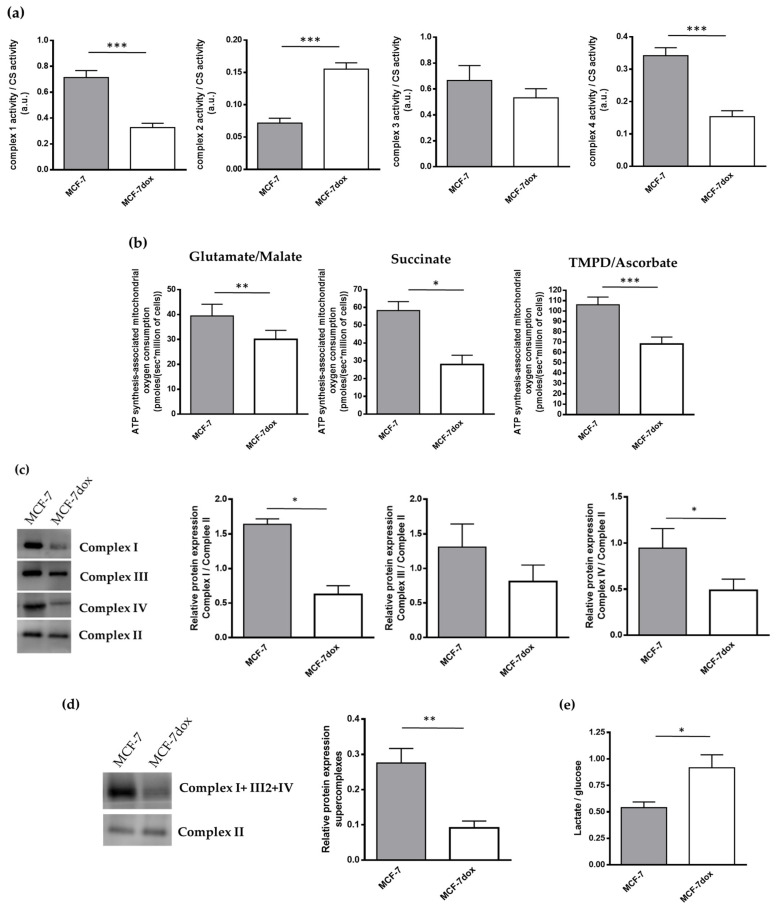
Increased glycolysis and dysfunctional mitochondria in DOX-resistant breast cancer cells. (**a**) Enzymatic activities of respiratory chain complexes 1, 2, 3, or 4 and citrate synthase were measured by spectrophotometry. Results are presented as the ratio of respiratory chain complexes activity to citrate synthase activity (*n* = 6); (**b**) mitochondrial oxygen consumption was measured by high-resolution respirometry. Experiment was carried out in the presence of glutamate + malate (complex I, *n* = 5), succinate (complex II, *n* = 6), or TMPD + ascorbate (complex IV, *n* = 9) and ADP as substrates; (**c**) holoenzyme for mitochondrial complex 1, 2, 3, and 4 was analyzed by BN Page. Results are presented as the ratios of complex 1, 3, or 4 holoenzyme content to complex 2 holoenzyme content (*n* = 6); (**d**) respiratory chain supercomplexes assembly was determined by Blue Native Page. Results were expressed as a ratio to complex II amount. (**e**) The levels of lactate production and glucose consumption were measured by spectrophotometry. Results are presented as the ratio of lactate production to glucose consumption (*n* = 21). The data are presented as mean ± SEM. * *p* < 0.05, ** *p* < 0.01, *** *p* < 0.005.

Maximal enzymatic activities of complex I and complex IV were 50% lower in MCF-7dox compared to MCF-7 (Figure 1a). By contrast, the activity of complex II was higher (2.5-fold). No change in complex III was observed (Figure 1a). Compared to MCF-7, mitochondrial oxygen consumption under an ATP synthesis condition in MCF-7dox with complex I- (glutamate/malate; −33%), complex II- (succinate; −52%), or complex IV-linked substrate (TMPD/ascorbate; −44%) (Figure 1b) was reduced, emphasizing a strong impact of complex I and complex IV dysfunction in MCF7-dox oxidative metabolism. Interestingly, we also evidenced a structural impairment of respiratory chain complexes in MCF7-dox cells since blue native PAGE analysis revealed a significant reduction of the quantity of complex I and complex IV holoenzyme (correctly assembled complex) in MCF-7dox compared to MCF-7 (−71% and −60%, respectively) (Figure 1c). However, no assembly intermediates (testifying to a complex subunits assembly defect) could be evidenced in MCF-7dox cells. In MCF7-dox, the number of supercomplexes I+III2+IV was also reduced compared to MCF-7 (−76%) (Figure 1d). Collectively, these data indicated that oxidative metabolism was altered in MCF-7dox. On the other hand, we found that the ratio of lactate production/glucose consumption was higher (+60%) in MCF-7dox than in MCF-7 (Figure 1e), suggesting that MCF-7dox cells had a predominant glycolytic metabolism.

### 2.2. ROS Production Is Increased and Is Associated to a Higher Expression of HIF-1 in DOX-Resistant Breast Cancer Cells

**Figure 2:**
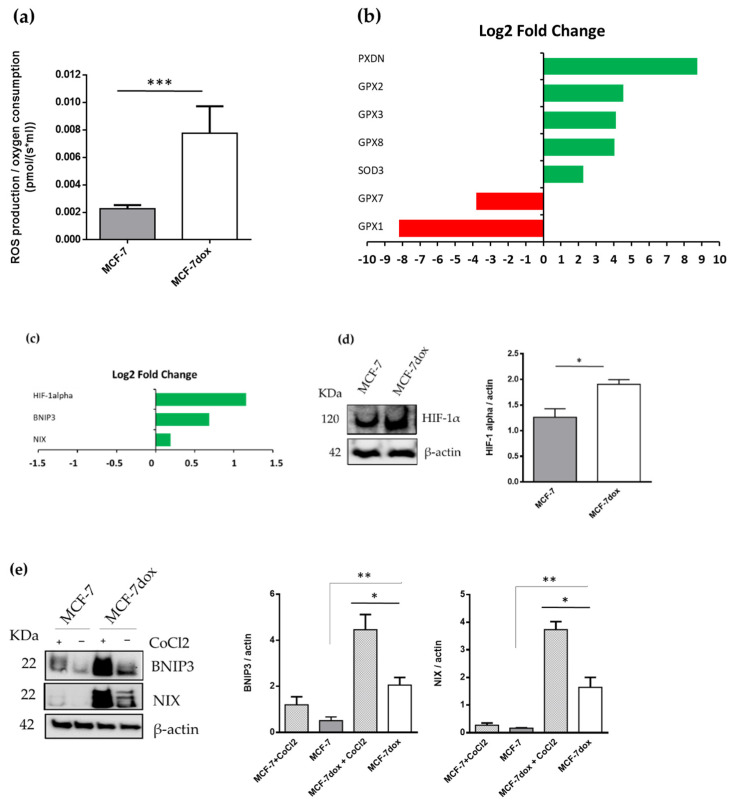
Mitochondrial ROS production is increased and is associated with a higher expression of hypoxia-inducible factor-1 in DOX-resistant breast cancer cells. (**a**) Mitochondrial ROS production was evaluated simultaneously with mitochondrial oxygen consumption by measuring the hydrogen peroxide-induced fluorescence of Amplex Red. Mitochondrial oxygen consumption was measured by high-resolution respirometry. Experiment was carried out in the presence of glutamate, malate, and ADP (*n* = 7); (**b**,**c**) gene expression analysis. Results are represented as Log2 Fold Change of MCF-7dox compared to MCF-7; (**d**) the level of HIF-1alpha and actin were measured by western blotting with specific antibodies. Densitometric analysis results are presented as the ratio of HIF-1alpha level to actin level (*n* = 4); (**e**) The level of BNIP3, NIX, and actin were measured by western blotting with specific antibodies. CoCl2 treated cells were used as a positive control for protein expression. Densitometric analysis results are presented as the ratios of BNIP3 and NIX levels to actin levels (*n* ≥ 5). The data are presented as mean ± SEM. * *p* < 0.05, ** *p* < 0.01, *** *p* < 0.005.

Our results showed that ROS production with respiratory chain complex I substrates was higher (×4) in mitochondria from MCF-7dox than in mitochondria from MCF-7 (Figure 2a). On the other hand, expression levels of mitochondrial antioxidant genes were higher in MCF-7dox than in MCF-7 (Figure 2b). It has been suggested that ROS play an important role in regulating the master transcription factor, HIF-1 alpha [18,19]. Our data showed that HIF-1 alpha was higher at both mRNA and protein levels in MCF-7dox than in MCF-7 in normoxia conditions (Figure 2c,d). Both BNIP3 and NIX are under the transcriptional regulation of HIF-1 alpha and increased HIF-1 alpha level could subsequently increase BNIP3 and NIX protein levels. We found that protein levels of both BNIP3 and NIX were higher in MCF-7dox than in MCF-7 (Figure 2e). In addition, mRNA levels of BNIP3 and NIX were also higher in MCF-7dox than in MCF-7 (Figure 2c). As expected, pretreatment of cells with CoCl2, a canonical hypoxia-mimetic agent known to stabilize HIF1α protein, was able to stimulate BNIP3 and NIX protein levels only in MCF-7dox (Figure 2e).

### 2.3. In the Basal Condition, There Is No Difference in Mitophagy between the Parental and the DOX-Resistant Breast Cancer Cell Line

**Figure 3:**
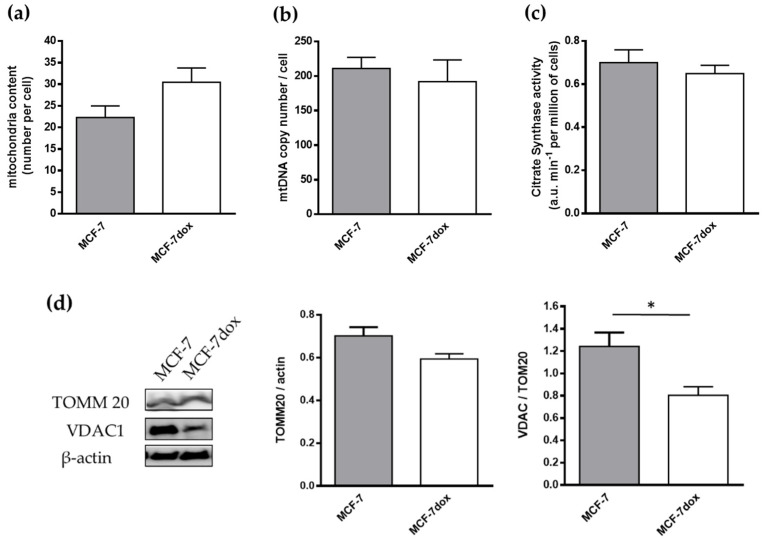
In the basal condition, there is no difference in mitophagy between the parental and the DOX-resistant breast cancer cell line. (**a**) Mitochondrial number was determined by transmission electron microscopy (*n* = 4, 10 cells/n); (**b**) mitochondrial DNA copy number was determined by quantitative PCR (*n* = 6); (**c**) maximal enzymatic activity of citrate synthase activity was measured by spectrophotometry (*n* = 6); (**d**) the levels of TOMM20 and VDAC-1 were measured by western blotting with specific antibodies. Densitometric analysis results are presented as the ratio of TOMM20 and VDAC-1 level to actin level (*n* = 5). The data are presented as mean ± SEM. * *p* < 0.05.

Active mitophagy is associated with a decrease in mitochondria content. Results showed that the number of mitochondria as measured by transmission electron microscopy was unchanged between MCF-7 and MCF-7dox (Figure 3a). No difference in the mitochondrial DNA content (mtDNA) and maximal activity of citrate synthase, other indicators of mitochondrial mass, was detected (Figure 3b,c). Furthermore, protein levels of TOMM20, a constitutively expressed outer membrane mitochondrial protein, were unchanged between the two cell lines (Figure 3d). Only the expression of VDAC-1 was lower in MCF-7dox than MCF-7 (Figure 3d). Collectively, these data suggested that mitochondrial mass was not different between MCF-7 and MCF-7dox and increased level of mitophagy proteins is not associated with a difference in mitophagy between the two cell lines in the basal condition.

### 2.4. Autophagolysosome Formation Is Activated by DOX Treatment in DOX-Resistant Breast Cancer Cells

**Figure 4:**
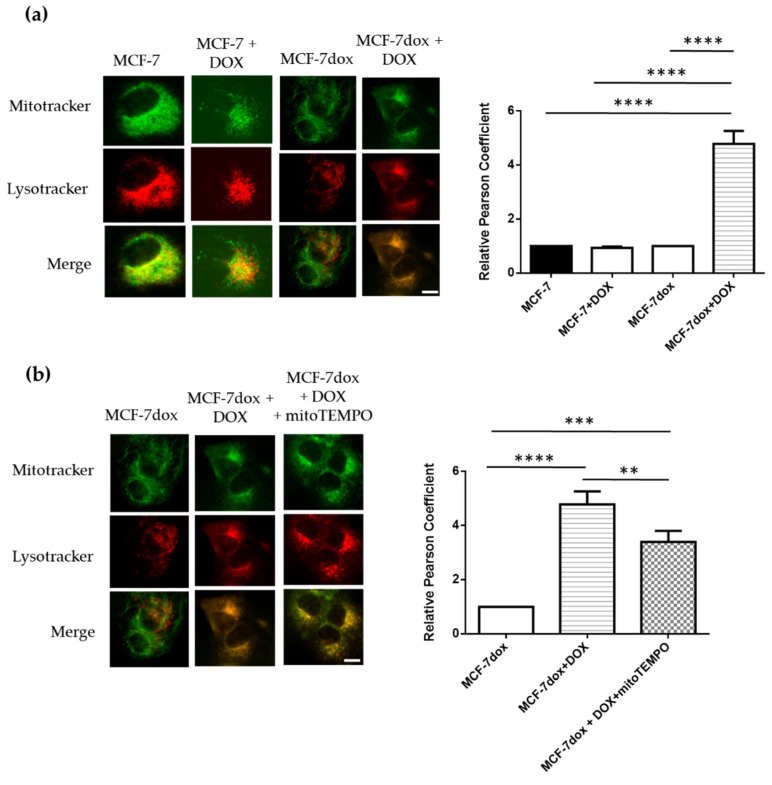
Autophagolysosome formation is activated by DOX treatment in DOX-resistant breast cancer cells. (**a**,**b**) Representative images of colocalization of lysosomes and mitochondria in MCF-7 and MCF-7dox (scale bar, 10 μm). Cells were co-stained with 60 nM MitoTracker Green and 100 nM LysoTracker Red for 45 min. Images of cells were acquired with fluorescence microscopy (Nikon eclipse Ti, Okolab) and Nikon’s NIS-Elements microscope imaging software. MCF-7 and MCF-7dox were treated with 100 nM and 30 µM of DOX, respectively (IC_50_ for each cell line), and 5 µM mitoTEMPO 24 h before the experiment. The colocalization of lysosomes and mitochondria was calculated using image-J software, and Pearson’s correlation coefficient (r) has been applied for quantifying colocalization. A total of 32–49 cells from five independent experiments. The data are presented as mean ± SEM. ** *p* < 0.01, *** *p* < 0.005, **** *p* < 0.0001.

We next studied mitophagic flux in MCF-7dox in response to DOX treatment by determining the colocalization of mitochondria within lysosomes (autophagolysosome formation). Mitochondria were labeled with MTG (green fluorescence), and lysosomes were labeled with red fluorescence. Mitochondrial and lysosomal co-localization was indicated by yellow fluorescence. Our results showed that the lysosomal staining intensity and especially the number of lysosomes colocalized with fragmented mitochondria were increased by DOX treatment in DOX-resistant breast cancer cells (MCF-7dox), while no effect of DOX was observed in MCF-7 parental cells (Figure 4a). Thus, colocalization of mitochondria with lysosomes was strongly increased by DOX treatment in MCF-7dox. Interestingly, pretreatment of MCF-7dox cells with the mitochondria-targeted antioxidant mitoTEMPO slightly reduced (−20%) DOX-induced colocalization of mitochondria with lysosomes (Figure 4b), suggesting that elimination of mitochondrial ROS can partly prevent mitophagy initiation. Collectively, these results suggested that autophagolysosome formation is induced by DOX treatment in MCF-7dox, and this effect is partly dependent on the increase in mitochondrial ROS level following DOX treatment.

## 3. Discussion

In the present study, we show that in DOX-resistant breast cancer cells, autophagolysosome formation is increased in response to DOX treatment. This effect is moderately ascribed to mitochondrial ROS production since induction of autophagolysosome formation is slightly diminished by the mitochondrial antioxidant mitoTEMPO. In addition, it seems that DOX-resistant breast cancer cells are prone to stimuli to initiate mitophagy since mitochondrial ROS production and HIF-1 alpha expression are higher in these cells in a basal condition. 

DOX resistance is an important cause of poor clinical outcomes in breast cancer patients. In the present study, we found that autophagolysosome formation, one of the steps of mitophagic flux, was initiated by DOX treatment in a resistant breast cell line model (MCF-7dox). This result suggests that mitophagy may mitigate the treatment-induced stress and participate in the DOX resistance of MCF-7dox. It has already been shown that activation of mitophagy could induce resistance to DOX in HCT8 colorectal cancer cells [14]. In addition, in DOX-sensitive breast cancer cell line models, DOX-mediated apoptosis is enhanced by the inhibition of autophagy/mitophagy with liensinine [15]. In the present study, we have specifically studied mitophagic flux by analyzing the colocalization of mitochondria/lysosomes. Interestingly, we also showed that DOX-induced accumulation of lysosomes within mitochondria was slightly attenuated by co-treatment with the mitochondrial antioxidant mitoTEMPO. It seems therefore that induction of mitophagic flux by DOX treatment is partly mediated by mitochondrial ROS. DOX is a redox-active compound that can react with several enzymes including mitochondrial complex I to generate semiquinone radical and high levels of ROS [8]. Collectively, our data suggest that mitophagic flux is induced in MCF-7dox via mitochondrial ROS-dependent mechanisms and could partly participate in DOX resistance in these cells. Further experiments on the different means of mitophagy and the effects of mitochondrial ROS are required to connect mitophagy and DOX resistance and ROS in breast cancer cells.

Another important finding in the present study is that mitochondrial ROS production and the HIF-1 alpha protein level were higher in MCF-7dox than in their DOX-sensitive counterparts (MCF-7) in basal conditions. Additionally, the levels of antioxidant enzymes such as GPX2, GPX3, and SOD3 are higher in MCF-7dox compared to MCF-7 cell lines, likely to counteract the increased level of ROS that is produced by MCF-7dox, thus preventing oxidative stress. ROS excess can induce the accumulation of defective mitochondria and, in turn, mitophagy to eliminate these dysfunctional mitochondria and thus prevent the initiation of the apoptotic cascade. In our MCF-7dox resistant cells, the increased level of ROS is associated with alteration of mitochondria function as evidenced by decreased activity of complex I and a decreased quantity of supercomplexes composed by complexes I, III, and IV. In addition, we found that MCF-7dox had a particular metabolic phenotype since glycolysis was increased. Surprisingly, our results showed that, in a basal condition, mitochondrial mass is not different between MCF-7 and MCF-7dox as evidenced by electron microscopy, citrate synthase activity, and protein content of mitochondrial biogenesis indicator, suggesting that mitophagy is not different between the two cell lines in basal condition. It is therefore not excluding that the default in mitochondrial oxidative phosphorylation we observed in MCF-7dox was not sufficient to signal for mitophagic clearance. Interestingly, we found that expression of HIF-1 alpha and BNIP3, a downstream regulator of HIF-1 alpha in hypoxia, is higher in MCF-7dox than in MCF-7. It is known that ROS play an important role in the regulation of HIF-1 alpha under normoxia and hypoxia [12]. In turn, HIF-1 alpha has been shown to induce mitophagy [13]. It seems, therefore, that an increased level of ROS and HIF-1 alpha in our MCF-7dox cells is not sufficient to stimulate mitophagy activation. However, the relationship between HIF-1 alpha, mitophagy, and ROS may be complex, since a previous study has demonstrated that loss of BNIP3 led to increased HIF-1 alpha levels and ROS production and conversely expression of exogenous BNIP3 markedly reduced HIF-1 alpha accumulation and led to mitophagy [20]. On the other hand, HIF-1 alpha is known to promote the metabolic switch and favor glycolysis [21,22]. Collectively, our data suggest a metabolic adaptation in MCF-7dox in order to induce resistance to DOX treatment. Both the higher mitochondrial ROS production and HIF-1 alpha expression allow MCF-7dox to be prone to mitophagy induction. While our results propose a role for ROS in DOX-induced autophagolysosome formation in DOX-resistant breast cancer cells, they do not provide either the underlying mechanism or the connection with HIF-1 alpha. In this respect, experiments to study the effects of DOX treatment on the production of mitochondrial ROS and the activity of HIF-1 alpha as well as experiments to demonstrate causal effect are required to clearly elucidate the mechanisms that are responsible for both mitophagy activation and DOX resistance of breast cancer cells. 

## 4. Materials and Methods

### 4.1. Cell Culture

MCF-7 (Michigan Cancer Foundation-7) and MCF-7dox cells (DOX resistant cells with an IC50 approximately 500 times greater than that of MCF-7 cells) (MCF-7dox line was a gift of Dr. K. Cowan, National Cancer Institute, Bethesda, USA) were cultured in a DMEM medium 4.5 g/L of glucose (Lonza, Levallois-Perret, France) supplemented with 5% FBS (Eurobio, Les Ulis, France). The cells were cultured at 37 °C with 5% CO_2_. The MCF-7dox cells were cultured in the presence of 1 μM of DOX in order to maintain their resistance. Before the experiments, DOX was withdrawn for 7 days.

### 4.2. Protein Extraction

The cell pellets were solubilized in lysis buffer (1% triton, 20 mM Tris base, 150 mM NaCl, 1 mM MgCl_2_, 1 mM CaCl_2_) supplemented with protease inhibitor (Protease Inhibitor Cocktail, Sigma, France). Samples were then incubated on ice for ten minutes followed by centrifugation at 11,000× *g* at 4 °C. The supernatant containing protein extracts was collected, and the proteins were quantified using BCA Protein Assay Reagent Kit (Life Technologies, Saint-Aubin, France). 

### 4.3. Electrophoresis Assay and Western Blotting

Protein extract was diluted in water, loading buffer (Thermo Fisher Scientific, Pierce, Illkirch, France), as well as NuPAGE 10X reducing and denaturing agent containing 500 mM dithiothreitol (DTT) (Thermo Fisher Scientific, Pierce). Proteins were separated on a 4–20% Mini-PROTEAN^®^ TGX™ Precast Protein Gel (Bio-Rad, Marnes-La-Coquette, France). The transfer was carried out under semi-dry conditions (Trans-Blot Turbo Transfer System, Bio-Rad, Marnes-La-Coquette, France) using a PVDF membrane. The proteins of interest were detected by overnight incubation with the primary specific antibodies at 4 °C under shaking condition. Antibodies used for experiments are as follows; anti-BNIP3 (44060S), anti-NIX (D4R4B), anti-TOMM20 (4272S), anti-Hif-1 alpha (D1S7W), and anti-VDAC-1 (ab34726, abcam, Cambridge, UK). All primary antibodies are purchased from Cell Signaling except VDAC-1. The next day, the membranes were washed twice with TBS 0.1% Tween and then incubated with the secondary antibody (Santa Cruz BioTechnology, Heidelberg, Germany) for 1 h at room temperature. The primary antibody solutions were diluted in TBS-Tween containing 4% BSA and those of secondary antibody in TBS-Tween containing 0.5% BSA. Finally, the membranes were washed three times for 5 min with TBS 0.1% Tween and bands were revealed using the Pierce ECL kit (Thermo Fisher Scientific, Illkirch, France). β-actin was used as an internal loading control (β-actin-HRP Santa Cruz). The images were taken using the ChemiDoc MP Imaging System (Bio-Rad, Marnes-La-Coquette, France). 

### 4.4. Fluorescence Microscopy

Mitophagy was studied by measuring the colocalization of mitochondria and lysosomes (autophagolysosome formation). Mitochondria were stained with MitoTracker™ Green FM dye (m7514, Thermo Fisher, Villebon-sur-Yvette, France) at a concentration of 100 nM. The staining of lysosomes was performed by using the 60 nM of LysoTracker™ Red DND99 (Invitrogen, Illkirch, France). Images were observed using fluorescence microscopy (Nikon eclipse Ti, Okolab, Rome, Italy) and Nikon’s NIS-Elements microscope imaging software. Pearson’s correlation coefficient (r) has been used for quantifying colocalization using the JACOP plugin for ImageJ. Autophagolysosome formation was studied following treatments with 30 µM DOX (Sigma, Quentin Fallavier, France) for 72 h or the combination of DOX and 1 µM mitoTEMPO (Sigma, Quentin Fallavier, France) for 24 h. 

### 4.5. Transmission Electron Microscopy for Mitochondrial Content Determination

Cells were fixed by incubation for 24 h in 4% paraformaldehyde, 1% glutaraldehyde in 0.1 M phosphate buffer (pH 7.2), and then washed in phosphate-buffered saline (PBS) and post-fixed by incubation with 2% osmium tetroxide for 1 h. Samples were then fully dehydrated in a graded series of ethanol solutions followed by a propylene oxide bath. The pre-impregnation step was made by a propylene oxide/Epon resin mixture and finally overnight in pure resin for impregnation of the samples. Cells were then embedded in Epon resin, which was allowed to polymerize for 48 h at 60 °C. Ultra-thin sections (90 nm) of these blocks were obtained with a Leica EM UC7 ultramicrotome (Wetzlar, Germany). Sections were deposited on gold grids and stained with 2% uranyl acetate and 5% lead citrate. Microscopy was performed using a JEOL 1011 transmission electron microscope. Images were analyzed using ImageJ software (NIH). Analyses were performed on 10 cells’ analysis for each section. This was repeated four times.

### 4.6. Mitochondrial Enzymatic Activities

The activities of the mitochondrial OXPHOS complexes were measured at 37 °C with a UVmc2 spectrophotometer (SAFAS, Monaco). The activity of the NADH ubiquinone reductase (C1), succinate ubiquinone reductase (C2), ubiquinol cytochrome C reductase (C3), cytochrome C oxidase (C4), and citrate synthase (CS) was measured according to standard methods [23].

### 4.7. Assembly of Mitochondrial Respiratory Chain Complexes and Supercomplexes

Complex I assembly was analyzed by Blue Native electrophoresis (BN Page) according to [24]. Briefly, mitochondria were isolated from cells pellets by differential centrifugation (10,000× *g*, 10 min, 4 °C) after digitonin permeabilization. Mitochondrial complexes were solubilized in Laurylmaltoside (3 g/g of proteins), and 50 µg of proteins were loaded and resolved in a native 4–16% acrylamide gel. Proteins were transferred to the PVDF membrane in a dry transfer apparatus (Bio-Rad, Marnes-La-Coquette, France). Membranes were immunoblotted with antibodies raised against NDUFB6 and NDUFS2 for complex 1, SDHA for complex 2 (used as loading reference), III core 2 for complex 3, and COX Va for complex 4 (1/1000e, Abcam, Cambridge, UK), and bands were revealed by chemiluminescence on an Odyssey apparatus (Li-Cor Biosciences). For supercomplexes detection, the same protocol was applied, except to larylmatoside solubilization, which was replaced with digitonin (3g/g of proteins).

### 4.8. ROS Production 

ROS production was studied via a novel approach of combining high-resolution respirometry using a 2 mL chamber OROBOROS Oxygraph 2K (Oroboros Instruments, Innsbruck, Austria) and fluorometric measurement of hydrogen peroxide (H_2_O_2_) production as previously described [25]. Briefly, the production of H_2_O_2_ was evaluated by measuring the hydrogen peroxide-induced fluorescence of 1 µM of Amplex Red (exc-em: 560-584 nm, Interchim, France) in the presence of horseradish peroxidase (10 UI/mL) and superoxide dismutase (40 UI/mL) at 37 °C under stirring. For simultaneous measurement of oxygen and H2O2 fluxes, cells (5 × 10^6^) were permeabilized by digitonin (8 µg per 10^6^ cells for MCF-7 and 10 µg per 10^6^ cells for MCF-7dox). Permeabilized cells were resuspended in the respiratory buffer (10 mM KH_2_PO_4_, 300 mM Mannitol, 10 mM KCl, 5 mM MgCl_2_, 1 mM EGTA, and 0.1% bovine serum albumin (BSA) fatty acid-free, pH 7.4). The experiment was carried out in the presence of glutamate (5 mM) and malate (5 mM) for complex I-driven mitochondrial oxygen consumption. The H_2_O_2_/O_2_ flux ratio is applied to evaluate the relative importance of H_2_O_2_ production at different respiratory states.

### 4.9. Glucose and Lactate Assay

Cells were seeded into 25 cm² flasks with 5 mL of culture media. To determine the ratio lactate production/glucose consumption, the media were collected after 24 h in contact with cells and assayed for glucose and lactate levels by using a glucose assay kit (Randox GL 2623, Randox Laboratories, Crumlin, UK) and lactate assay kit (kit Olympus OSR6193, Olympus Life Science Research Europa, O’Callaghan’s Mills, Ireland) according to the manufacturer’s instructions. The values were analyzed by the OD values. Glucose consumption and lactate production were calculated based on the standard curve, normalized to the cell number and time.

### 4.10. Citrate Synthase Activity on Mitochondrial Extracts

Citrate synthase activity was measured by using the coupled reaction as follows: Acetyl-CoA+oxaloacetate+H_2_O → citrate+CoA-SH (colorimetric reaction: CoA-SH+DTNB → TNB+CoA-S-S-TNB). Citrate synthase activity was determined by measuring the appearance of the yellow product (TNB), which absorbed at 412 nm. The reaction medium consisted of 0.1 mM DTNB, 4% Triton X-100, 0.6 mM acetyl CoA, and mitochondrial proteins samples (10 µg). Twenty microliters of oxalacetate (10 mM) were added to start the reaction. The absorbance changes were measured every 20 s over 3 min at 412 nm to determine the citrate synthase activity. All assays were carried out at 37 °C. The activity of citrate synthase was expressed as nmol/(min*million of cells).

### 4.11. Gene Expression Analysis 

Gene expression was measured on 4 RNA extracts from MCF7 and MCF7-dox cell pellets using the Ion AmpliSeq™ Transcriptome Human Gene Expression Kit (Thermo Fisher Scientific) according to the manufacturer protocol. Briefly, cDNA was synthetized from 10 ng of total RNA using the SuperScript™ VILO™ cDNA Synthesis Kit (Thermo Fisher Scientific). Up to 20,000 genes were amplified with a single multiplexed targeted Ampliseq panel (Ion AmpliSeq™ Human Gene Expression Core Panel, Thermo Fisher Scientific). After ligation of adaptators and barcodes, libraries were loaded on Ion 540™ Chips using an Ion Chef™ apparatus (Thermo Fisher Scientific). Libraries were sequenced on the Ion GeneStudio S5 Next Generation Sequencer, and gene count was realized thanks to the ampliSeqRNA pipeline from Ion Torrent Suite (Thermo Fisher Scientific). Enrichment and differential expression analysis were performed with the IDEP 9.1 workflow (http://bioinformatics.sdstate.edu/idep/, accessed on 20 July 2021.) using the DESEQ2 package for differentially expressed gene determination.

### 4.12. Mitochondrial DNA Content Analysis

mtDNA copy number was measured in MCF-7 and MCF-7dox as described [26]. Briefly, total DNA was extracted from frozen cell pellets, and two mitochondrial genes (ND4 and COX1) were amplified by quantitative PCR. The Ct values were determined and compared to a standard curve with a known mtDNA copy number (from 10^7^ to 10^3^). The results were normalized with the amount of two nuclear genes (betazmicroglobulin and glycerol 3 phosphate dehydrogenase) to obtain the mtDNA copy number per cell.

### 4.13. Statistical Analysis

Results were expressed as mean ± SEM. Data were analyzed using graphPad Prism. Significance between groups was determined by one-way ANOVA with Sidak’s multiple comparisons test or by a Mann–Whitney test for two groups and Kruskal–Wallis for multiple comparisons. *p* < 0.05 was considered to be statistically significant.

## 5. Conclusions

In conclusion, in this study, we found that (1) in DOX-resistant breast cancer cells, autophagolysosome formation is increased in response to DOX treatment; (2) this effect is moderately ascribed to mitochondrial ROS production; and (3) DOX-resistant breast cancer cells seem to be prone to stimuli to induce mitophagy since mitochondrial ROS production and HIF-1 alpha expression are higher in these cells in the basal condition. Overall, our findings provide a potential rationale for approaching mitophagy induction as a potential biomarker to monitor resistance of breast cancer cells to DOX.

## Data Availability

The data are presented in this study.

## Abbreviations

ATP	Adenosine triphosphate
BNIP3	BCL2/adenovirus E1B 19 kDa protein-interacting protein 3
NIX	BCL2/adenovirus E1B 19 kDa protein-interacting protein 3 Like
DOX	Doxorubicin
HIF-1 alpha	Hypoxia-inducible factor-1 alpha
OXPHOS	Oxidative phosphorylation
ROS	Reactive oxygen species
TOMM20	Translocase of outer mitochondrial membrane 20
VDAC-1	Voltage-dependent anion-selective channel 1
